# Ancestral APOBEC3B Nuclear Localization Is Maintained in Humans and Apes and Altered in Most Other Old World Primate Species

**DOI:** 10.1128/msphere.00451-22

**Published:** 2022-11-14

**Authors:** Ashley A. Auerbach, Jordan T. Becker, Sofia N. Moraes, Seyed Arad Moghadasi, Jolene M. Duda, Daniel J. Salamango, Reuben S. Harris

**Affiliations:** a Department of Biochemistry and Structural Biology, University of Texas Health San Antonio, San Antonio, Texas, USA; b Institute for Molecular Virology, University of Minnesota – Twin Cities, Minneapolis, Minnesota, USA; c Department of Biochemistry, Molecular Biology, and Biophysics, University of Minnesota – Twin Cities, Minneapolis, Minnesota, USA; d Department of Microbiology and Immunology, University of Minnesota – Twin Cities, Minneapolis, Minnesota, USA; e Department of Microbiology and Immunology, Stony Brook University, Stony Brook, New York, USA; f Howard Hughes Medical Institute, University of Texas Health San Antonio, San Antonio, Texas, USA; University of Michigan Medical School

**Keywords:** APOBEC3B, DNA cytosine deamination, host-pathogen adaptation, primate innate immunity, subcellular localization

## Abstract

APOBEC3B is an innate immune effector enzyme capable of introducing mutations in viral genomes through DNA cytosine-to-uracil editing. Recent studies have shown that gamma-herpesviruses, such as Epstein-Barr virus (EBV), have evolved a potent APOBEC3B neutralization mechanism to protect lytic viral DNA replication intermediates in the nuclear compartment. APOBEC3B is additionally unique as the only human DNA deaminase family member that is constitutively nuclear. Nuclear localization has therefore been inferred to be essential for innate antiviral function. Here, we combine evolutionary, molecular, and cell biology approaches to address whether nuclear localization is a conserved feature of APOBEC3B in primates. Despite the relatively recent emergence of *APOBEC3B* approximately 30 to 40 million years ago (MYA) in Old World primates by genetic recombination (after the split from the New World monkey lineage 40 to 50 MYA), we find that the hallmark nuclear localization of APOBEC3B shows variability. For instance, although human and several nonhuman primate APOBEC3B enzymes are predominantly nuclear, rhesus macaque and other Old World primate APOBEC3B proteins are clearly cytoplasmic or cell wide. A series of human/rhesus macaque chimeras and mutants combined to map localization determinants to the N-terminal half of the protein with residues 15, 19, and 24 proving critical. Ancestral APOBEC3B reconstructed from present-day primate species also shows strong nuclear localization. Together, these results indicate that the ancestral nuclear localization of APOBEC3B is maintained in present-day human and ape proteins, but nuclear localization is not conserved in all Old World monkey species despite a need for antiviral functions in the nuclear compartment.

**IMPORTANCE** APOBEC3 enzymes are single-stranded DNA cytosine-to-uracil deaminases with beneficial roles in antiviral immunity and detrimental roles in cancer mutagenesis. Regarding viral infection, all seven human APOBEC3 enzymes have overlapping roles in restricting virus types that require DNA for replication, including EBV, HIV, human papillomavirus (HPV), and human T-cell leukemia virus (HTLV). Regarding cancer, at least two APOBEC3 enzymes, APOBEC3B and APOBEC3A, are prominent sources of mutation capable of influencing clinical outcomes. Here, we combine evolutionary, molecular, and cell biology approaches to characterize primate APOBEC3B enzymes. We show that nuclear localization is an ancestral property of APOBEC3B that is maintained in present-day human and ape enzymes, but not conserved in other nonhuman primates. This partial mechanistic conservation indicates that APOBEC3B is important for limiting the replication of DNA-based viruses in the nuclear compartment. Understanding these pathogen-host interactions may contribute to the development of future antiviral and antitumor therapies.

## INTRODUCTION

The apolipoprotein B mRNA editing enzyme catalytic-like polypeptide 3 (APOBEC3) family of single-stranded (ss)DNA cytosine deaminases forms a distinct arm of the mammalian antiviral innate immune response (1–3). Humans encode seven APOBEC3 (A3) enzymes, which have either one (A3A, A3C, and A3H) or two structurally conserved deaminase domains (A3B, A3D, A3F, and A3G) (4, 5). These seven enzymes have elicited a wide range of overlapping antiviral activities through both deaminase-dependent and deaminase-independent mechanisms (6). A3 enzymes have been shown to restrict retroviruses such as HIV-1 and HTLV-1, as well as several classes of double-stranded (ds)DNA viruses such as papillomaviruses (HPV), polyomaviruses (BK- and JC-PyV), and herpesviruses (EBV, KSHV, and HSV-1) (1, 2, 7–9). Retroviruses have obvious ssDNA replication intermediates (cDNA) that help explain susceptibility to A3 enzymes, whereas dsDNA viruses may expose sufficient ssDNA during lytic replication and/or transcription where structures such as lagging-strand intermediates and R-loop structures, respectively, may contribute to ssDNA exposure.

In addition to virus restriction activity, a hallmark of antiviral factors such as A3 enzymes is that susceptible viruses have evolved equally potent counterdefenses (1, 2, 7, 10). A recent example is gamma-herpesviruses, such as EBV and KSHV, which utilize the viral ribonucleotide reductase (RNR) large subunit to neutralize nuclear A3B (1, 11–13). Specifically, the viral RNR binds A3B, blocks DNA deaminase activity, and causes relocalization from the nucleus to the cytoplasm (1, 11–13). Structural studies have demonstrated that the binding interface is several hundred square-angstroms and includes direct sequestration of the loop regions of A3B required for ssDNA deamination (14). More recent studies have demonstrated mechanistic conservation, such that A3B binding and relocalization are mediated by gamma-herpesviral RNRs but only if the virus has adapted for replication in a host species with A3B (13). As further evidence for an ancient and ongoing battle between A3B and primate gamma-herpesviruses, A3B binding and relocalization activities have been lost in herpesviruses that infect New World primate species, which naturally lack the entire gene (13).

Human A3B is unique because it is the only human DNA deaminase family member to localize constitutively to the nuclear compartment (15–18). Original studies mapped nuclear localization determinants to the N-terminal half of A3B (15, 18). A series of chimeric constructs between human A3B and cytoplasmic family members (A3G, A3D) further whittled down this activity to two regions (17). The first region is comprised of residues 1 to 30 of human A3B, including D19 and E24, and the second region is proposed to include amino acids 79 to 109.

Nuclear localization is predicted to be essential for A3B’s role in restricting the replication and pathogenesis of viruses with lifecycles in the nucleus. However, the mechanism of A3B nuclear import/retention and the impact of A3B localization on viral restriction activities are not well characterized. To address whether nuclear localization is an evolutionarily conserved feature of primate A3B enzymes, we assembled a panel of A3B expression constructs from a wide variety of nonhuman primate species for characterization of subcellular localization and ssDNA deaminase activities. Each enzyme was tagged with enhanced green fluorescent protein (EGFP) and imaged in human and nonhuman primate cell lines using fluorescence microscopy. Human and ape A3B enzymes exhibit strong nuclear localization, whereas other nonhuman primate A3B enzymes localize cell wide or in the cytoplasm. Using human and rhesus macaque A3B as comparators, we constructed A3B chimeras that further mapped the localization determinants to the amino(N)-terminal domain (NTD), identifying residues 15, 19, and 24 as essential. Lastly, using the full panel of primate A3Bs, we reconstructed an ancestral A3B sequence and observed that the ancestral A3B enzyme also exhibits strong nuclear localization. Together, these results indicate that the nuclear localization of A3B is conserved from an ancestral primate to present day humans and apes, whereas this property has become altered in many nonape primate species. This suggests that different evolutionary pressures may have existed for different primate groups, such as human/apes versus New World monkeys (NWM), resulting in the distinct A3B subcellular localization patterns described here for modern enzymes.

## RESULTS

### Primate A3B proteins show diversity in subcellular localization.

**FIG 1:**
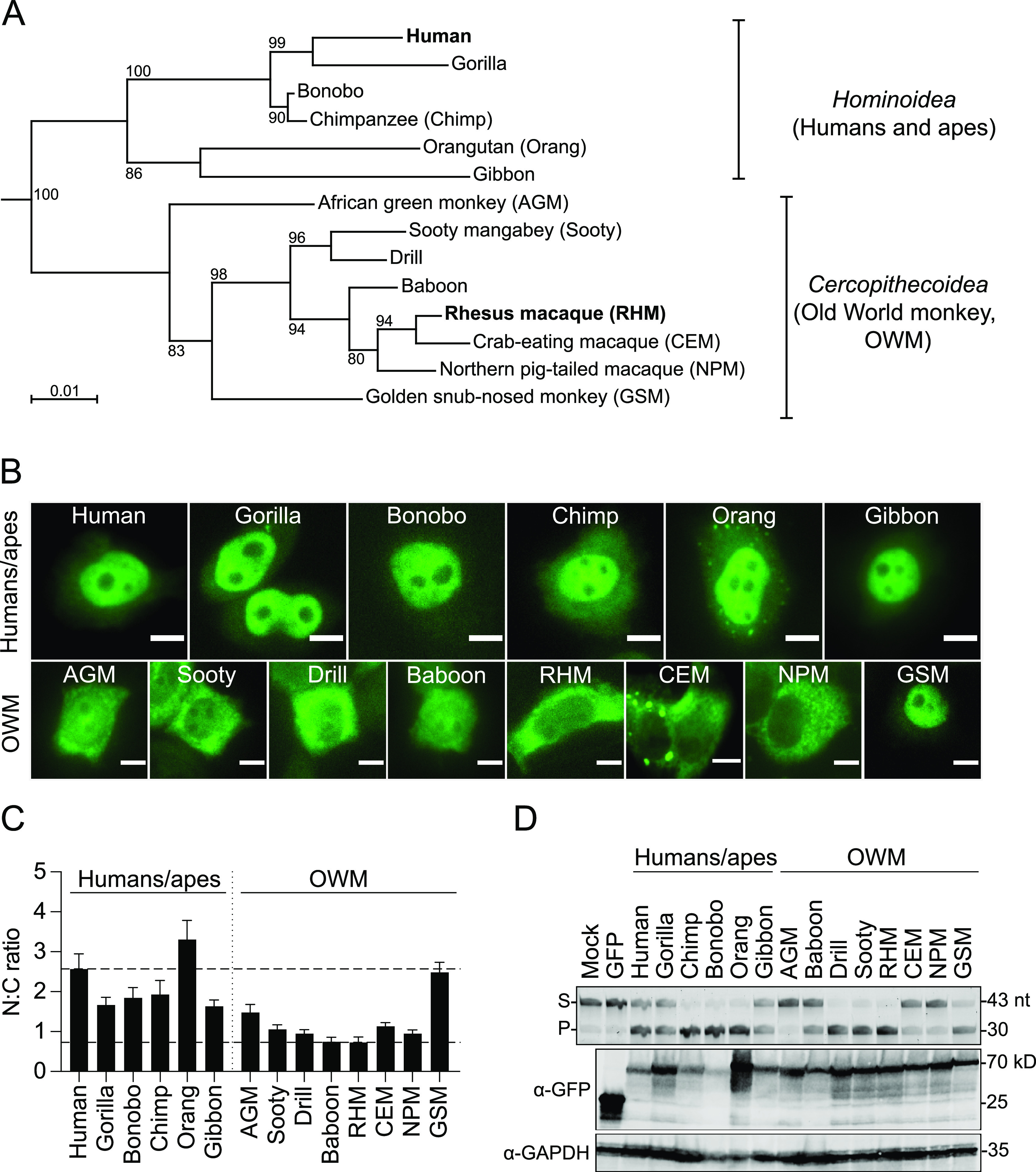
Diversity in subcellular localization of primate A3B proteins. (A) Phylogeny of primate *A3B* coding sequences used in this study. Nucleotide sequences were aligned using ClustalOmega and the phylogenetic tree was generated using PhyML in SeaView. Numbers indicate branch support from 100 bootstraps. (B) Representative fluorescence microscopy images of the indicated primate A3B-EGFP constructs expressed in HeLa (scale = 10 μm). (C) Quantification of N:C ratios for each A3B-EGFP construct (mean +/− SEM of >20 cells per condition). To facilitate comparisons, the upper dotted line shows the mean ratio for human A3B (nuclear) and the lower dotted line for rhesus macaque A3B (cytoplasmic). (D) DNA deaminase assay and immunoblots showing activity and expression levels of the indicated primate A3B-EGFP constructs in 293T. The ssDNA substrate (S) has a single C target motif, which leads to a single faster-migrating product (P). A3B-EGFP constructs were detected with an anti-EGFP antibody and anti-GAPDH provided a loading control.

To test the hypothesis that A3B nuclear localization is conserved, we first assembled a comprehensive panel of primate *A3B* cDNA sequences from public databases (see Fig. S1 in the supplemental material). The relatedness of the *A3B* coding sequences was assessed by generating a phylogenetic tree (Fig. 1A). The relative positioning of each primate *A3B* sequence agrees well with a time tree of primate genomes with two major branches, one for *Hominoidea* (apes and humans) and a second for *Cercopithecoidea* (Old World monkeys) (Fig. S2). The time tree is a public knowledge forum that incorporates molecular sequence and clock data to infer and resolve phylogenetic relationships over deep time (19, 20). The time tree is robust and likely reflects actual evolutionary history. Thus, differences between these two trees are expected because a single gene tree has more variability and, in the instance of *A3B* as a positively selected antiviral factor, is likely to manifest even more variation (13, 21, 22). For instance, in the gene-based tree, human *A3B* shows the highest sequence homology with gorilla *A3B*, and African green monkey (AGM) *A3B* (subfamily *Cercopithecinae*) appears more basal than golden snub-nosed monkey (GSM) (subfamily *Colobinae*) (Fig. 1A, Fig. S2). In reality, humans and chimpanzees/bonobos are more closely related than humans and gorillas, and the GSM is the most diverged animal in this group of primates (Fig. 1A, Fig. S2) (23).

10.1128/msphere.00451-22.1 FIG S1 Primate and ancestral A3B amino acid sequence alignment. Alignment of primate and ancestral A3B amino acid sequences. Protein accession numbers are listed next to each species for reference. For ease of comparison, the human A3B sequence (blue) and rhesus macaque A3B (yellow) are highlighted with ancestral A3B listed at the bottom. Amino acid alignment starts at residue 1 through the full length of A3B (residue 382). Dots indicate residues that are conserved with the human sequence. Download FIG S1, EPS file, 1.4 MB. Copyright © 2022 Auerbach et al. 2022 Auerbach et al. https://creativecommons.org/licenses/by/4.0/ This content is distributed under the terms of the Creative Commons Attribution 4.0 International license.

10.1128/msphere.00451-22.2 FIG S2 Phylogenetic tree of primate species. Phylogeny of primate species used in this study generated using TimeTree and pruned using interactive Tree of Life (iTOL). Numbers above dashed vertical lines represent time in millions of years ago (MYA). Human and rhesus macaque are bolded for ease of comparison. Primate groups are indicated with the bars on the right-hand side (*Hominoidea* and *Cercopithecoidea*). Download FIG S2, EPS file, 1.0 MB. Copyright © 2022 Auerbach et al. 2022 Auerbach et al. https://creativecommons.org/licenses/by/4.0/ This content is distributed under the terms of the Creative Commons Attribution 4.0 International license.

To study the subcellular localization of these primate A3B proteins, each cDNA was cloned into an expression construct with a C-terminal flexible linker and EGFP and then transfected into HeLa for visualization. Using fluorescence microscopy, we imaged this panel of primate A3B-EGFP proteins and quantified their nucleocytoplasmic localization (representative images in Fig. 1B and quantification in Fig. 1C). As expected, A3B proteins from apes, including human, gorilla, bonobo, chimpanzee (chimp), orangutan (orang), and gibbon exhibited predominantly nuclear localization. However, to our surprise, subcellular localization of primate A3Bs is variable in more distantly related primate species (i.e., Old World monkeys, OWM). Notably, A3B’s from multiple OWM species exhibit distinctly cytoplasmic localization, including the proteins from sooty mangabey (SMM), drill, rhesus macaque (RHM), crab-eating macaque (CEM), and northern pig-tailed macaque (NPM). In contrast, A3B from AGM and baboon exhibits whole-cell distributions, whereas A3B from GSM is distinctly nuclear. Despite notable diversity in subcellular localization patterns of A3B in OWM species, the distinctly nuclear localization of human/ape A3B enzymes and GSM A3B combines to suggest that nuclear localization may be an ancestral property.

To assess the integrity of each construct, whole-cell extracts were fractionated by SDS-PAGE and immunoblots were done to show that each protein is expressed at the expected molecular weight (Fig. 1D). Expression levels also showed some variability, bonobo versus orangutan A3B, for instance, despite identical amounts of plasmid DNA in each transient transfection and similar amounts of protein loading. Therefore, ssDNA C-to-U deaminase assays were also done to assess the activity of each enzyme. Whole-cell extracts were treated with RNase and incubated with an ssDNA containing a single C substrate. In the presence of uracil DNA glycosylase (UDG), uracil excision yields an abasic site, and upon treatment with sodium hydroxide the substrate (S) oligonucleotide is cleaved, and the product (P) migrates further on an acrylamide gel. Nearly all primate A3B proteins (except AGM A3B) elicit catalytic activity relative to untransfected cell extracts (mock) and EGFP-transfected cell extracts (Fig. 1D). The lack of AGM A3B catalytic activity may be explained by the presence of residue N314 compared to the conserved D314 residue in loop 7 of the A3B catalytic domain (Fig. S1). Substitutions of loop 7 amino acid residues of A3 catalytic domains have been shown to dramatically alter enzymatic activity (24, 25).

### The NTD of human and RHM A3B enzymes determines subcellular localization.

**FIG 2:**
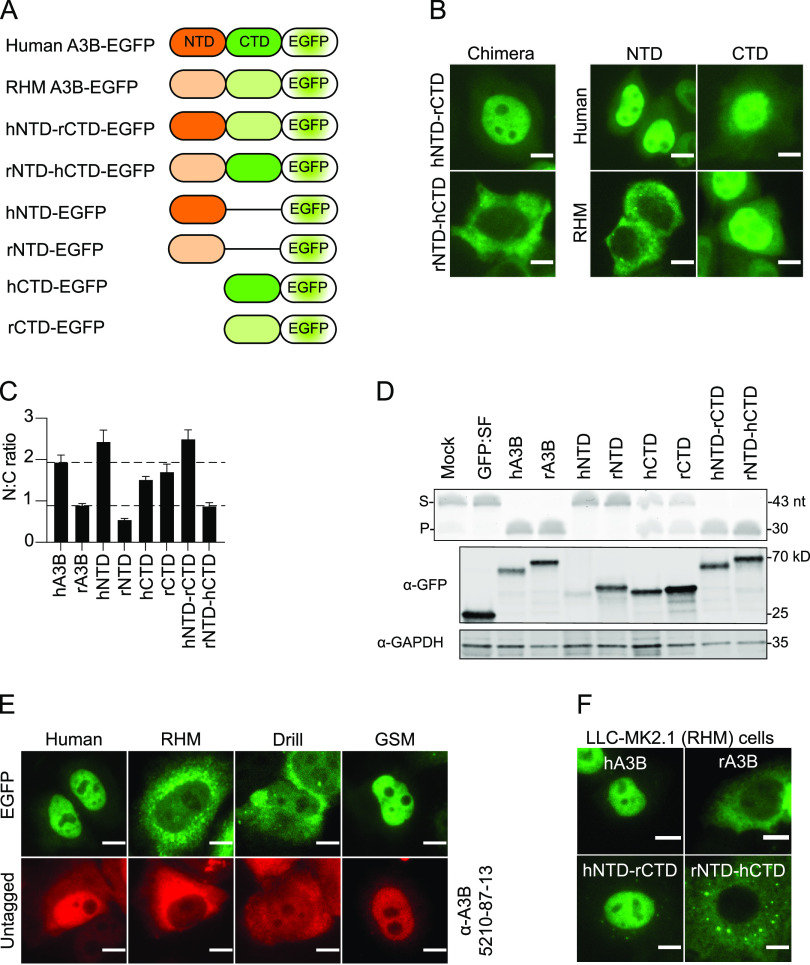
The NTD governs A3B localization to the nucleus or cytoplasm. (A) Construct schematics. (B) Representative fluorescence microscopy images of the indicated constructs expressed in HeLa (scale = 10 μm). (C) Quantification of N:C ratios for each A3B-EGFP construct (mean +/− SEM of >20 cells per condition). To facilitate comparisons, the upper dotted line shows the mean ratio for human A3B (nuclear) and the lower dotted line for rhesus macaque A3B (cytoplasmic). (D) DNA deaminase assay and immunoblots showing activity and expression levels of the indicated primate A3B-EGFP constructs in 293T. The ssDNA substrate (S) has a single C target motif, which leads to a single faster-migrating product (P). A3B-EGFP constructs were detected with an anti-EGFP antibody and anti-GAPDH as a loading control. (E) Representative fluorescence microscopy images comparing the indicated EGFP-tagged (green) and untagged (red) A3B constructs in HeLa (scale = 10 μm). To detect untagged A3B (red), cells were fixed and permeabilized, then probed for A3B using a custom monoclonal antibody (rabbit anti-human A3B MAb 5210-87-13). (F) Representative fluorescence microscopy images of the indicated A3B-EGFP proteins expressed in LLC-MK2.1 (scale = 10 μm).

Given that human and RHM A3B show different subcellular localization patterns (nuclear and cytoplasmic, respectively), these two proteins were used to confirm whether the NTD or the carboxy-terminal domain (CTD) governs this activity. Previous work showed that the NTD of human A3B is responsible (17, 18, 26). To reconfirm this result for human A3B and potentially extend it to RHM A3B, we first generated a set of reciprocal chimeras, transfected these constructs into HeLa, and found that subcellular localization tracks with the NTD of each species’ protein (construct schematics in Fig. 2A; representative images in Fig. 2B left; quantification in Fig. 2C). Second, we generated individual constructs for the NTD and CTD of each species and transfected them into HeLa. This approach showed that the NTD-EGFP of human A3B is distinctly nuclear and the NTD-EGFP of RHM A3B is distinctly cytoplasmic, whereas the CTD-EGFP constructs from each species are distributed cell wide (construct schematics in Fig. 2A; representative images in Fig. 2B right; quantification in Fig. 2C). All constructs are expressed at the predicted kilodalton size and, as expected, only CTD-containing constructs elicit catalytic activity (Fig. 2D).

To confirm that the subcellular localization determinants of primate A3B enzymes are exclusive to the NTD, we generated a full panel of NTD-EGFP constructs and analyzed localization patterns in transfected HeLa. These experiments showed that the localization of primate NTD-EGFP constructs largely mirrors the localization of the corresponding full-length primate A3B-EGFP proteins (images in Fig. S3A and Fig. 1B, respectively). To confirm that the localization patterns of these primate A3B proteins are not perturbed by the addition of the EGFP tag, expression constructs encoding untagged human, drill, RHM, and GSM A3B were transfected into HeLa and immunofluorescence microscopy was done using an anti-A3B monoclonal antibody (no. 5210-87-13; Fig. 2E, red). Notably, human and GSM untagged A3B show predominantly nuclear localization, whereas drill and RHM A3B still exhibit predominantly cytoplasmic localization. These results take advantage of the fact that the C-terminal antibody binding epitope is conserved and further demonstrate that localization of these A3B proteins is independent of epitope tag or cell type (i.e., subcellular localization is an intrinsic property of A3B). We also found that the subcellular localization of representative primate NTD-EGFP proteins in RHM LLC.MK2.1 is similar to the same constructs expressed in HeLa (Fig. S3B and Fig. 2F, respectively). EGFP-tagged human-RHM chimeric proteins localized in an NTD-dependent manner in RHM LLC.MK2.1, further indicating that subcellular localization is a general property of A3B and unlikely to be an artifact of an individual cell line or species (Fig. 2F). We next wanted to confirm that A3B does not contain a canonical nuclear localization signal (NLS) or nuclear export signal (NES). We treated HeLa transfected with EGFP-tagged human and RHM A3B with the CRM1/XPO1 inhibitor Leptomycin B (LMB) or the IMPORTIN-α/β inhibitor ivermectin (IVM). Human AID-EGFP served as a positive control due to its well-characterized NES and known sensitivity to LMB treatment (27–31). We found that neither human nor RHM A3B localization is altered by LMB or IVM treatment, whereas human AID-EGFP is confined to the nuclear compartment by LMB treatment (Fig. S3C).

10.1128/msphere.00451-22.3 FIG S3 A3B NTD localization mirrors full-length A3B and is independent of cell type. (A) Representative fluorescent microscopy images of the indicated NTD-EGFP constructs expressed in HeLa (scale = 10 μm). NTD-EGFP construct is shown in schematic. Images are grouped as human/apes (top) and OWM (bottom). (B) Representative fluorescent microscopy images of the indicated NTD-EGFP constructs expressed in LLCMK2.1 (scale = 10 μm). NTD-EGFP construct is shown in schematic. (C) Representative fluorescent microscopy images of the indicated constructs tagged with EGFP expressed in HeLa (scale = 10 μm). Cells were treated with 12.5 nM Leptomycin B (CRM1/NES inhibitor), 25 μM ivermectin (IMPORTIN-alpha/NLS inhibitor), or vehicle control for two hours prior to fixation and imaging. Download FIG S3, TIF file, 1.3 MB. Copyright © 2022 Auerbach et al. 2022 Auerbach et al. https://creativecommons.org/licenses/by/4.0/ This content is distributed under the terms of the Creative Commons Attribution 4.0 International license.

### Residues 1 to 25 of A3B NTD control nucleocytoplasmic localization.

**FIG 3:**
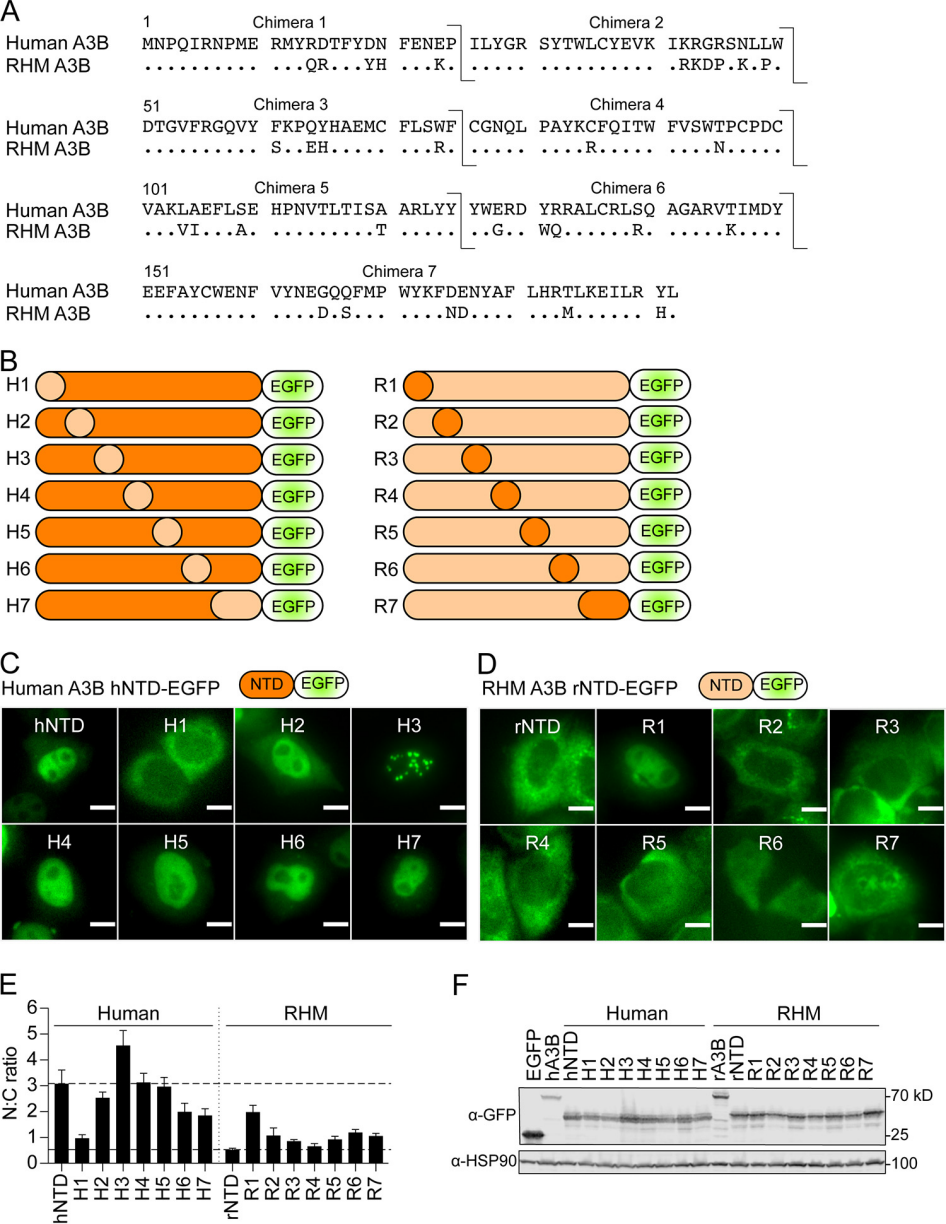
The amino-terminal 25 residues of A3B control subcellular localization. (A) Amino acid alignment of human and RHM A3B NTD by ClustalOmega. Arrows indicate locations of chimera junctions used to generate 25 residue sequential chimeras between human and rhesus A3B NTD-EGFP. Chimera junction points were selected at areas of residue conservation between human and rhesus macaque A3B. (B) Construct schematics of A3B chimeras. Each construct consists of A3B NTD fused to EGFP. Constructs H1 to H7 indicate a human A3B backbone (dark orange) with 25 amino acid segments changed to rhesus macaque residues (light orange). Constructs R1-R7 indicate a rhesus macaque A3B backbone (light orange) with 25 amino acid segments changed to human residues (dark orange). (C and D) Representative fluorescence microscopy images of human (C) and RHM (D) 25 amino acid A3B NTD-EGFP chimeras expressed in HeLa (scale = 10 μm). Chimeras H1 and R1 exhibit changed localization compared to the wild type, indicating the importance of residues 1 to 25 in A3B subcellular localization. (E) Quantification of N:C ratios for each NTD-EGFP chimeric construct (mean +/− SEM of >20 cells per condition). To facilitate comparisons, the upper dotted line shows the mean ratio for human A3B (nuclear) and the lower dotted line for rhesus macaque A3B (cytoplasmic). (F) Immunoblots showing activity and expression levels of the indicated NTD-EGFP chimeric constructs in 293T. NTD-EGFP constructs were detected with an anti-EGFP antibody, and anti-HSP90 provided a loading control. All constructs are well expressed and observed at the expected molecular weight.

To determine the residues responsible for subcellular localization of primate A3B, we first generated sequential chimeras in 25 amino acid increments of the human and RHM A3B NTD (amino acid alignment shown in Fig. 3A; construct schematics in Fig. 3B). Second, we transfected the parent A3B NTD constructs and chimeras into HeLa and used fluorescence microscopy to determine subcellular localization. We found that the key determinants of human A3B NTD localization to the nucleus and RHM A3B NTD localization to the cytoplasm map within the first 25 amino acids of each of these proteins (Fig. 3C and D; quantification in Fig. 3E). Specifically, whereas human A3B NTD-EGFP is nuclear, replacing amino acids 1 to 25 with those of rhesus A3B changed its localization to the cytoplasm (human chimera 1, H1). The converse is apparent for RHM A3B NTD-EGFP encoding the first 25 amino acids of human A3B (rhesus chimera 1, R1), which changed to a predominantly nuclear localization. Unexpectedly, human chimera 3 (H3, replacing residues 50 to 75 with the corresponding rhesus A3B residues) was still nuclear but with a distinctly punctate pattern. The other chimeras showed largely similar localization to their corresponding wild-type A3B NTD-EGFP (see quantification in Fig. 3E). The converse chimera (rhesus chimera 3, R3) remained cytoplasmic without a noticeable change to its general distribution. Immunoblots showed that all the A3B NTD-EGFP chimeric proteins expressed similarly in 293T (Fig. 3F). These combined results indicate that the first 25 amino acids of A3B NTD contain the major determinants for subcellular localization.

### Residues 15, 19, and 24 are determinants of primate A3B nucleocytoplasmic localization.

**FIG 4:**
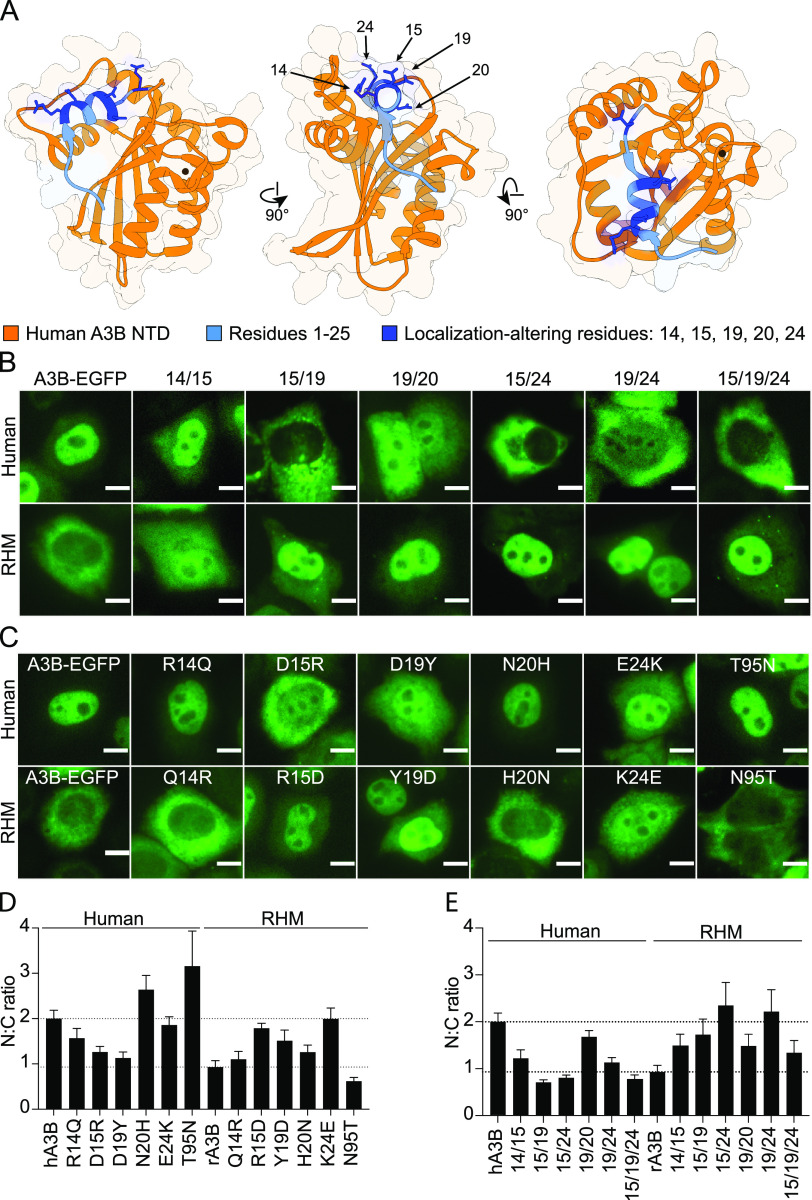
Residues 15, 19, and 24 in primate A3B modulate subcellular localization. (A) Ribbon model of the crystal structure of human A3B NTD (orange; PDB code 5TKM) shown in three different poses. Residues 1 to 25 are shown in light blue, and residues 14, 15, 19, 20, and 24 are shaded dark blue with solvent-exposed side chains shown. (B) Representative fluorescence microscopy images of human (top) and rhesus (bottom) A3B-EGFP multiple residue substitution mutants in HeLa (scale = 10 μm). (C) Representative fluorescence microscopy images of human (top) and rhesus (bottom) A3B-EGFP single residue substitution mutants in HeLa (scale = 10 μm). (D) Quantification of N:C ratios for each single residue substitution mutant A3B-EGFP construct (mean +/− SEM of >20 cells per condition). To facilitate comparisons, the upper dotted line shows the mean ratio for human A3B and the lower dotted line for rhesus macaque A3B. (E) Quantification of N:C ratios for each double or triple residue substitution mutant A3B-EGFP construct (mean +/− SEM of >20 cells per condition). To facilitate comparisons, the upper dotted line shows the mean ratio for human A3B (nuclear) and the lower dotted line for rhesus macaque A3B (cytoplasmic).

To determine which of the first 25 amino acids contribute to nucleocytoplasmic localization, we compared the sequences of human and RHM A3B and focused on the 5 residues that differ within this region (Fig. 3A). We noted that all 5 of these residues are clustered along alpha helix 1 and loop 1 of the human A3B NTD structure (PDB code 5TKM) (32). Specifically, the side chains of residues 14, 15, 19, 20, and 24 are solvent exposed (dark blue) and shown in the context of residues 1 to 25 (lighter blue) and the larger human A3B NTD (orange; Fig. 4A). We observed that two of the residues (14 and 20) are positioned relatively flat compared to the other three (19, 33, 34), which have side chains projecting upward away from the rest of the NTD structure and into the solvent. These 5 residues in full-length human A3B were changed in all pairwise combinations to the corresponding RHM residues, and the resulting mutant constructs were transfected into HeLa and imaged using fluorescence microscopy (representative images in Fig. 4B, top panel, and quantification in Fig. 4D). We observed that combinations of 15/19, 15/24, and 19/24 caused normally nuclear human A3B to become cytoplasmic. The 15/19/24 triple mutant was also similarly cytoplasmic. Likewise, the reciprocal double and triple mutant combinations caused normally cytoplasmic RHM A3B to become predominantly nuclear (representative images in Fig. 4B, lower panel, and quantification in Fig. 4E). Other double mutant combinations also perturbed the cytoplasmic localization of RHM A3B suggesting that this property may be more sensitive to disruption. As an additional control, the double and triple mutants expressed in 293T at levels like those of the parental human and RHM A3B-EGFP constructs by immunoblotting of whole-cell extracts (Fig. S4A).

10.1128/msphere.00451-22.4 FIG S4 Model structure of RHM A3B NTD shows residues 15, 19, and 24 on surface. (A) Immunoblots showing expression of double and triple amino acid substitution A3B-EGFP proteins in 293T. Human and RHM A3B-EGFP constructs were detected with an anti-EGFP antibody and anti-HSP90 provided a loading control. (B) Ribbon model of the RoseTTAFold models of RHM A3B NTD (gray, residues1 to 192) shown in three poses. Residues1 to 25 are shown in light green and residues 15, 19, and 24 are shown in dark green with the amino groups shown. (C) Depiction of human A3B NTD overlayed with the RHM A3B NTD models with same coloring as in Fig. 4A and Fig. S4B. Download FIG S4, TIF file, 1.4 MB. Copyright © 2022 Auerbach et al. 2022 Auerbach et al. https://creativecommons.org/licenses/by/4.0/ This content is distributed under the terms of the Creative Commons Attribution 4.0 International license.

To determine which single residue(s) contribute to nucleocytoplasmic localization of human and rhesus A3B, we generated all 5 reciprocal single amino acid substitution mutants and assessed subcellular localization in HeLa (representative images in Fig. 4C and quantification in Fig. 4E). We also tested reciprocal changes at residue 95, which is the only amino acid difference between human and RHM A3B within a previously implicated surface region (17). Single amino acid substitutions at position 95 had little effect. Interestingly, however, reciprocal changes at residues 15, 19, and 24 clearly perturb human A3B nuclear localization, although none of the single changes was as strong as the double and triple mutant phenotypes described above. Likewise, reciprocal changes of residues 15, 19, and 24 in RHM A3B caused the protein to become more nuclear with the single amino acid changes at 15 and 19 triggering human A3B-like nuclear localization. We observe that residues 15, 19, and 24 in combination are required for A3B subcellular localization. Single mutation of residues 15, 19, and 24 can alter the localization of human and rhesus macaque A3B but is insufficient for complete relocalization.

To enable comparisons with the actual structure of human A3B NTD (PDB code 5TKM) (32), RoseTTAFold was used to generate a structural model of RHM A3B NTD (Fig. S4B). Five predicted models of RHM A3B NTD are superimposed and displayed as ribbon structures (light gray) in three poses with residues 1 to 25 highlighted in light green and residues 14, 15, 19, 20, and 24 in dark green. Again, residues 15, 19, and 24 are positioned facing upward and more exposed to solvent relative to the core of the structure. The actual structure of human A3B NTD and the RoseTTAFold-predicted RHM A3B NTD are very similar with an RMSD value of 0.76 Å (Fig. S4C).

Previous work has shown that many *A3* genes, including *A3A*, *A3D*, *A3G*, and *A3H*, are evolving under positive selection, with variable residues/regions often helping to define sites of direct interaction with viral proteins (33, 35–39). To ask whether the subcellular localization region defined above in A3B is under positive selection, mixed effects model of evolution (MEME) was used to infer site-specific selection across our panel of primate A3B sequences (40). Multiple sites appeared under modest positive selection in both the NTD and CTD (Fig. S5). Among the positively selected residues are one important for interaction with primate lentiviral Vif proteins (residue 128), multiple amino acids implicated in A3B neutralization by herpesviral ribonucleotide reductases (residues 209 and 245/247/249 in two distinct loop regions), and two residues shown to be important for A3G subcellular localization (residues 14 and 15) (14, 18, 41). Taken together with the results above for human and rhesus A3B, it is possible that at least one undefined virus may antagonize A3B restriction activity by perturbing subcellular localization.

10.1128/msphere.00451-22.5 FIG S5 MEME analysis of A3B shows that residues 14 and 15 are under positive selection. (A) Positive selection inference by MEME depicting *P*-values for per-site positive selection. *P*-values between 1.0 and 0 are shown. Residues 14, 15, 128, 209, and 245 are highlighted. (B) Positive selection inference by MEME depicting *P*-values for per-site positive selection. *P* values between 0.3 and 0.0 are shown. Residues 14, 15, 128, 209, and 245 are highlighted. Download FIG S5, EPS file, 1.3 MB. Copyright © 2022 Auerbach et al. 2022 Auerbach et al. https://creativecommons.org/licenses/by/4.0/ This content is distributed under the terms of the Creative Commons Attribution 4.0 International license.

### Ancestral A3B is nuclear and catalytically active.

**FIG 5:**
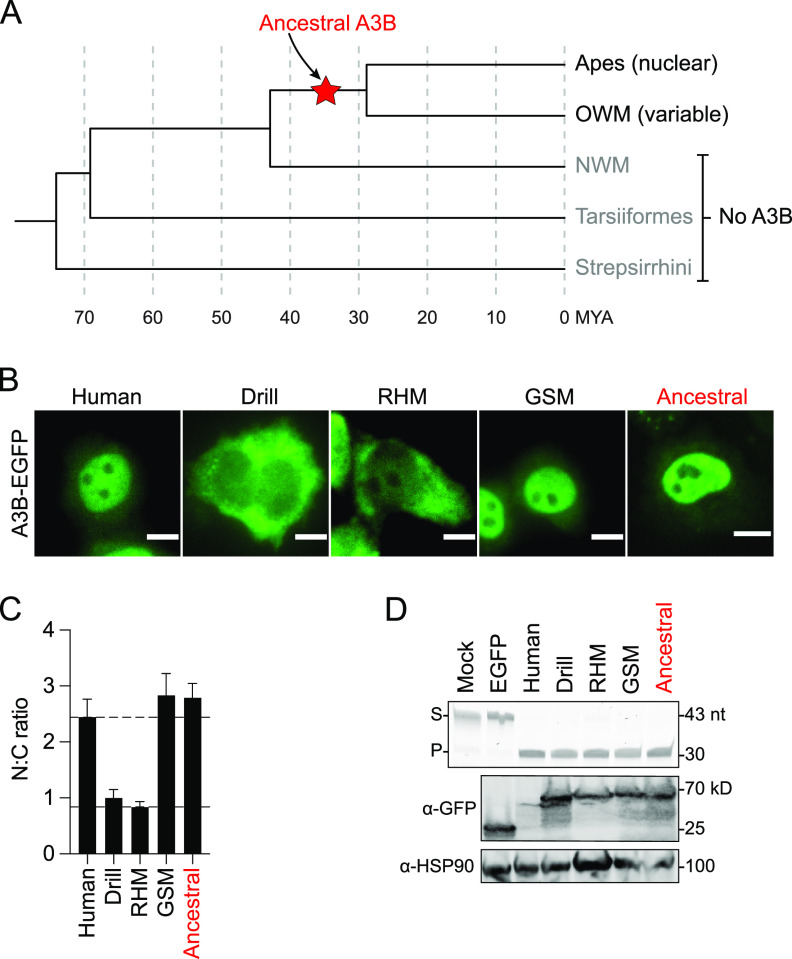
Ancestral A3B exhibits nuclear localization. (A) Phylogeny of primate families depicting the generation of an ancestral *A3B* gene (red star) prior to the split of apes and OWM (and after the split of NWM lineages). (B) Representative fluorescence microscopy images of the indicated constructs expressed in HeLa (scale = 10 μm). (C) Quantification of N:C ratios for each A3B-EGFP construct (mean +/− SEM of >20 cells per condition). To facilitate comparisons, the upper dotted line shows the mean ratio for human A3B (nuclear) and the lower dotted line for rhesus macaque A3B (cytoplasmic). (D) DNA deaminase assay and immunoblots showing activity and expression levels of the indicated primate A3B-EGFP constructs in 293T. The ssDNA substrate (S) has a single C target motif, which leads to a single faster-migrating product (P). A3B-EGFP constructs were detected with an anti-EGFP antibody and anti-HSP90 provided a loading control.

Comparative studies inferred that the ancestral *A3B* gene emerged by unequal crossing-over between ancient *A3* genes (5, 42). Taken together with studies demonstrating that *A3B* is specific to apes and OWM and not found at all in NWM (here and refs. 13, 43), one can infer that the ancestral *A3B* gene emerged sometime after the evolutionary split of OWM and NWM, but before the more recent split into humans/apes and OWM subgroups (Fig. 5A; refs. 23, 44–46). This means that ancestral *A3B* originated between 30 and 40 million years ago (MYA) and has been maintained in most humans/apes and OWM species to present day.

We therefore hypothesized that the ancestral A3B enzyme would exhibit nuclear localization that is maintained in humans and apes but altered in many (but not all) lineages of OWM (e.g., GSM A3B is nuclear). Because primate A3B subcellular localization is not conserved and may have been driven by evolutionary pressures inferred from positive selection, a computationally derived ancestral protein sequence was constructed using graphical representation of ancestral sequence predictions (GRASP) (47, 48). The resulting sequence was created as a gene block, cloned upstream of EGFP, and expressed as an A3B-EGFP fusion protein for localization studies in HeLa (ancestral protein sequence included at bottom of Fig. S1 alignments). As shown above, human A3B and GSM A3B are nuclear, and RHM and drill A3B are cytoplasmic (Fig. 5B with quantification in Fig. 5C). In comparison, ancestral A3B exhibits a nuclear localization phenotype easily as strong as human and GSM A3B proteins (Fig. 5B with quantification in Fig. 5C). Furthermore, ancestral A3B is expressed comparably to the other primate A3B constructs and elicits similarly strong single-stranded DNA C-to-U editing activity (Fig. 5D). Together these results suggest that the original primate A3B emerged as an antiviral factor localized to the nuclear compartment and that this activity has been maintained in the human/ape lineage but not in the OWM lineage.

## DISCUSSION

A3B is an antiviral enzyme known to restrict viruses by inducing mutations in viral DNA genomes and DNA intermediates. An important feature of a virus restriction factor, such as A3B, is that its subcellular localization enables spatial compatibility with the pathogenic target. Therefore, we hypothesized that nuclear localization of A3B, as observed in humans, would be a conserved feature of A3B across primate species. Here, we show that A3B nuclear localization is indeed conserved among the human and apes, but its localization shows surprising range of variation in OWM species. Using nuclear human and cytoplasmic rhesus macaque A3B as comparators, we mapped the determinants of A3B subcellular localization to residues 15, 19, and 24 of the NTD. We also found that reconstructed ancestral A3B exhibits nuclear localization, which suggests that A3B nuclear localization is maintained in humans and apes, whereas A3B subcellular localization is less constrained in OWM species.

The reason(s) for the maintenance of A3B nuclear localization in humans and apes is currently unknown but potentially due to selective pressures imposed by viral pathogens that replicate in this cellular compartment. For instance, a strong emerging candidate is herpesviruses, which have coevolved with vertebrates and are estimated to have emerged >100 MYA (49, 50). Recent studies have revealed that present-day gamma-herpesviruses such as EBV and KSHV have a potent and specific A3B neutralization mechanism (1, 11–14). The large subunit of the viral ribonucleotide reductase (RNR subunit BORF2 in EBV and ORF61 in KSHV) specifically binds to loop regions within the A3B deaminase domain (A3B CTD), inhibits A3B DNA deamination activity, and causes the dramatic relocalization of A3B from the nuclear to the cytoplasmic compartment. The interaction is specific to A3B as the most closely related A3 enzymes are bound less efficiently (A3A) or not at all (A3G) (13, 14). Importantly, EBV lacking the large RNR subunit becomes susceptible during lytic replication to A3B-catalyzed deamination, manifests C/G-to-T/A hypermutations, and shows significantly lower infectivity (11). Moreover, recent analyses of a comprehensive panel of viral RNRs strongly suggest that the interaction with A3B is conserved among gamma-herpesviruses that infect humans/apes/OWM species and is completely lacking in gamma-herpesviruses that infect NWM species (13). This striking association strongly suggests that the origination of A3B as a restriction factor in ancestral primates could have forced ancient herpesviruses to adapt to neutralize this enzyme or face elimination. The variability in OWM A3B localization is presently unexplained but it is easy to envisage the involvement of other viruses that imposed different and perhaps even stronger selective pressures (e.g., an ancestral virus that replicated and assembled in the cytoplasm).

**FIG 6:**
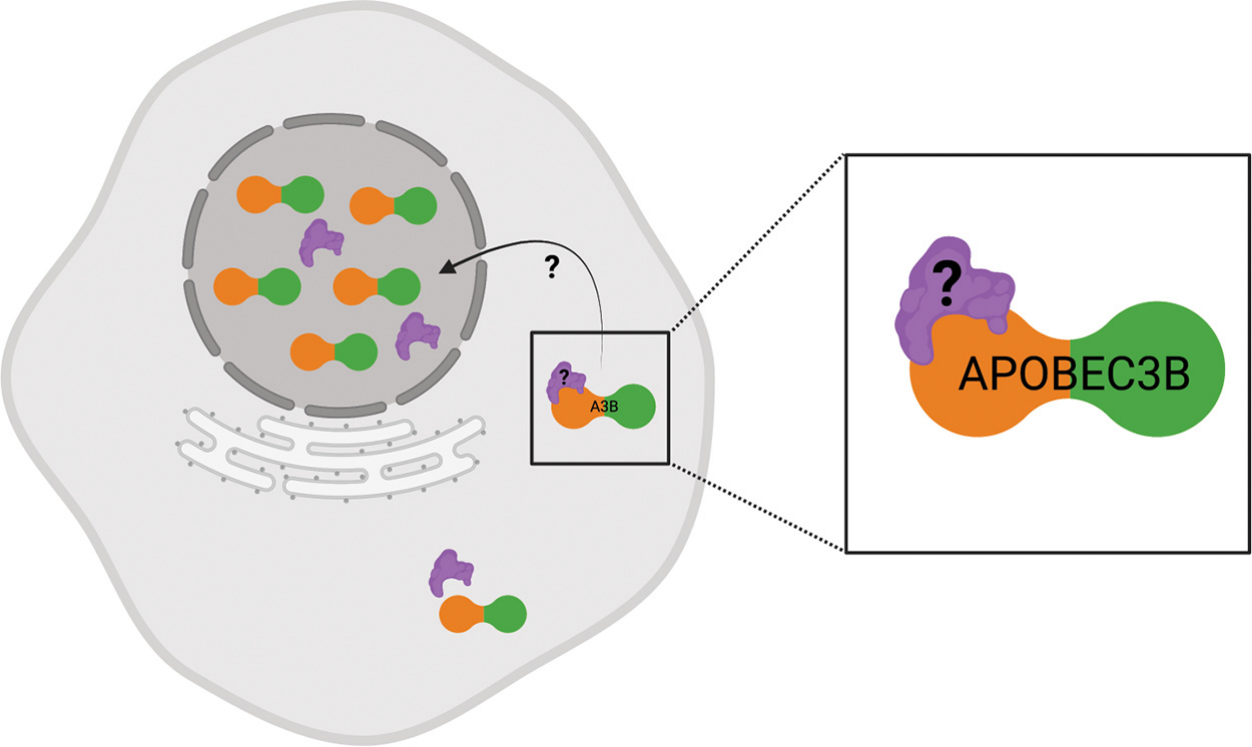
Model for A3B nuclear import. Schematic of putative A3B nuclear import mechanism. A currently unidentified factor (protein or protein complex) provides a bridge between A3B and IMPORTIN-α, allowing for import of A3B into the nucleus. The nuclear import factor (purple) binds to human/ape A3B (NTD in orange and CTD in green) in the cytoplasm and allows for import through the nuclear pore. Residues 15, 19, and 24 are important for A3B nuclear localization and predicted to be essential for this interaction. Created with BioRender.com.

The *cis*-determinants of A3B nuclear localization include residues 15, 19, and 24, and it is striking that all three of these residues cluster on the same solvent-exposed surface of the protein (α1-loop 1 region in Fig. 4A and Fig. S4). This physical clustering strongly suggests a direct interaction with an as-yet-unidentified factor that promotes nuclear localization. Previous studies suggested that A3B enters the nucleus through an IMPORTINα-dependent mechanism, despite the absence of a canonical nuclear localization signal (17, 18, 26). Moreover, a six-amino-acid substitution mutant of human A3B engineered to be defective for binding to RNA is still able to localize to the nuclear compartment (51). These results combine to suggest that the currently unidentified factor may be a protein (or protein complex) that provides a bridge between A3B and IMPORTIN-α or another import protein (depicted in Fig. 6). This unidentified protein may bind and import A3B into the nucleus through interaction with residues 15, 19, and 24 and additional interactions with import machinery. This raises the question as to whether different factors are responsible for cytoplasmic or cell-wide localization of primate A3B enzymes or whether these alternative states may be governed by less specific interactions with RNA, self-oligomerization, and/or other cellular components.

Elucidating the mechanism of A3B subcellular localization is paramount in understanding A3B’s activity as a restriction factor and cancer mutagen. Importantly, the subcellular location/compartment in which A3B accumulates in infected cells has the potential to dictate the spectrum of restriction-susceptible viruses and accordingly influence pathogenesis. We predict that A3B has evolved in human and ape species as a restriction factor against viruses with lifecycles in the nucleus, such as herpesviruses. In addition, A3B is associated with specific mutational patterns in several types of human cancers and its access to the nucleus (and therefore chromosomal DNA) is crucial to this activity (52–55). We postulate that primate species with cytoplasmic A3B may be less subject to A3B-associated carcinogenesis over their lifetimes and that increased cytoplasmic A3B is protective against cancer. We also theorize that there would be no detectable A3B-signature mutations in NWM tumors because these primate species lack *A3B*. If this prediction holds true, cancers with A3B-catalyzed mutation signatures may be confined to humans, apes, and a subset of OWM lineages. Future work is needed to identify the mechanisms of human and ape A3B nuclear localization, determine the breadth of A3B activity in primate tumor development, and discover additional viral pathogens subject to A3B restriction.

## MATERIALS AND METHODS

### Plasmid DNA constructs.

A3B sequences were collected from the NCBI, EMBL Ensembl, or UCSC ENCODE databases. The cDNA sequences are available upon request and the corresponding protein sequences and GenBank accessions are shown in Fig. S1. All plasmid DNA constructs were generated by conventional molecular biology cloning methods. Primate A3B sequences were ordered as IDT gBlock double-stranded DNA products encoding a chimeric intron between codons 241 and 242 (homologous to the exon 5 and 6 splice junction), blunt-end cloned into pJET1.2 to isolate sequence confirmed products, and subsequently cloned into a pcDNA5/TO expression vector with a C-terminal EGFP tag (Invitrogen no. V103320). NTD/CTD domain swaps of human and RHM A3B were generated by overlapping PCR (PCR) and cloned into pcDNA5/TO (hNTD-rCTD, rNTD-hCTD). Isolated human and RHM NTD and CTD constructs were generated by PCR and subcloned back into pcDNA5/TO. Untagged primate A3B constructs were generated by PCR and subcloned back into pcDNA5/TO. Human and RHM A3B-NTD chimeras were generated by overlapping PCR and cloned into the pcDNA5/TO vector: H1 (rNTD_1-25_-hNTD_26-192_), H2 (hNTD_1-25_-rNTD_26-50_-hNTD_51-192_), H3 (hNTD_1-50_-rNTD_51-75_-hNTD_76-192_), H4 (hNTD_1-75_-rNTD_76-100_-hNTD_101-192_), H5 (hNTD_1-100_-rNTD_101-125_-hNTD_126-192_), H6 (hNTD_1-124_-rNTD_125-150_-hNTD_151-192_), H7 (hNTD_1-150_-rNTD_151-192_), R1 (hNTD_1-25_-rNTD_26-192_), R2 (rNTD_1-25_-hNTD_26-50_-rNTD_51-192_), R3 (rNTD_1-50_-hNTD_51-75_-rNTD_76-192_), R4 (rNTD_1-75_-hNTD_76-100_-rNTD_101-192_), R5 (rNTD_1-100_-hNTD_101-125_-rNTD_126-192_), R6 (rNTD_1-124_-hNTD_125-150_-rNTD_151-192_), R7 (rNTD_1-150_-hNTD_151-192_). Human and RHM single, double, and triple amino acid residue mutations were generated by site-directed mutagenesis (hA3B_R14Q_, hA3B_D15R_, hA3B_D19Y_, hA3B_N20H_, hA3B_E24K_, hA3B_T95N_, hA3B_RD14/15QR_, hA3B_DD15/19RY_, hA3B_DE15/24RK_, hA3B_DN19/20YH_, hA3B_DE19/24YK_, hA3B_DDE15/19/24RYK_, rA3B_Q14R_, rA3B_R15D_, rA3B_Y19D_, rA3B_H20N_, rA3B_K24E_, rA3B_N95T_, rA3B_QR14/15RD_, rA3B_RY5/19DD_, rA3B_RK5/24DE_, rA3B_YH19/20DN_, rA3B_YK19/24DE_, rA3B_RYK15/19/24DDE_). Human AID-EGFP plasmid has been described (26, 56). All PCRs were completed using Phusion High-Fidelity DNA polymerase (ThermoFisher no. F530L). PCR primers were purchased from Integrated DNA Technologies and are available on request. All plasmid DNA constructs were sequence confirmed by Sanger sequencing (Azenta/Genewiz, NJ, USA).

### Bioinformatics, phylogenetics, and structural modeling.

A phylogenetic tree was generated based on the DNA sequences of the primate A3B enzymes (Fig. S1). Briefly, coding DNA sequences (CDS) of primate *A3B* genes were aligned using ClustalOmega (57) in Seaview5 (58) and a phylogenetic tree estimated using PhyML (34) with 100 bootstraps. The OWM time tree was generated for all OWM species from TimeTree5 (20, 59) and pruned to species of interest using the interactive Tree of Life (iTOL) (60). Positive selection analysis was performed using MEME (40) via DataMonkey (61). The ancestral A3B protein sequence was generated using graphical representation of ancestral sequence predictions (GRASP) (47, 48). The structural model of RHM A3B NTD was generated by submitting the amino acid sequence of residues 1 to 192 for deep learning-based model prediction by RoseTTAFold (62). Additional structural information used here includes the X-ray structure of human A3B-NTD (PDB code 5TKM) (32) and RHM A3B NTD models are available upon request.

### Cell culture.

HeLa, HEK293T, and LLCMK2.1 cell lines were derived from laboratory stocks. All cells for this study were incubated in high glucose DMEM (Gibco no. 11995073), supplemented with 1% penicillin/streptomycin (Gibco no. 15140-122), 1% GlutaMAX (Gibco no. 35050079), and 10% fetal bovine serum (Gibco no. 26140-079). Plasmid DNA transfections were performed using 3 μg polyethylenimine (PEI, Polysciences, Inc. no. 23966) transfection reagent per 1 μg DNA in 100 μL Opti-MEM reduced-serum media (Gibco no. 31985062), combined, vortexed, incubated for 20 min, and then added dropwise to preadhered cells in complete growth medium.

### Fluorescence microscopy.

Approximately 25,000 HeLa cells were plated in growth media into a flat-bottomed 24-well tissue culture plate (Sigma-Aldrich no. Z707805). The cells were incubated overnight to allow for adherence. After 24 h, the cells were cotransfected with 200 ng of each A3B expression construct and 80 ng of an mCherry expression construct. At 48 h posttransfection, cells were fixed with 4% paraformaldehyde in phosphate-buffer saline (PFA-PBS). Each well was incubated at room temperature in 500 μL PFA-PBS per well for 15 min, then washed 3 times with PBS. Each well was then incubated with DAPI at room temperature with gentle rocking for 10 min and washed 3 times with PBS. Cells were imaged at ×20 magnification on a Cytation 1 Cell Imaging Multimode Reader (BioTek) with the following LED cubes: DAPI (377/447), EGFP (469/525), and Texas Red (586/647). Images were processed using Fiji/ImageJ (63) to subtract background and quantified using a CellProfiler v4.2.1 pipeline (64). The pipeline identified individual nuclei, cell boundaries, and measured fluorescent intensity of the segmented nuclear and cytoplasmic compartments. DAPI staining was used to identify the nuclear compartment and mCherry was used to identify whole-cell area. The cytoplasmic compartment was determined in CellProfiler by subtracting the nuclear compartment from the whole-cell area. To quantify the subcellular localization of each A3B enzyme, mean EGFP intensity was measured in the nucleus and cytoplasm. All experiments were performed in biological triplicate with quantification calculated from representative images taken from a single set of experiments with a minimum of 20 cells per condition. Subcellular localization was reported as the nuclear-to-cytoplasmic (N:C) ratio of mean EGFP intensity.

### Immunofluorescence microscopy.

To ensure that tagging APOBEC3B with EGFP does not impact the subcellular localization, a subset of untagged primate A3Bs were imaged by immunofluorescence microscopy. Untagged human, drill, RHM, and golden snub-nosed monkey were cloned, transfected, and imaged per the protocol described above with the addition of a permeabilization and antibody incubation steps. After fixing with 4% PFA-PBS, cells were permeabilized by incubating with 0.25% Triton X-100 in PBS for 10 min. Then, each well was blocked for 1 h at room temperature with blocking buffer (3% BSA in PBS). An antibody developed by the Harris laboratory was used to detect untagged A3B at a concentration of 1:250 diluted in blocking buffer and incubated for 1 h at room temperature (rabbit antihuman A3B MAb 5210-87-13) (65). Then, each well was washed with three times with PBS and incubated for 1 h with secondary antibody (goat antirabbit AlexaFluor594, Invitrogen; diluted 1:1000 in blocking buffer). After washing 3 times with PBS, cells were incubated with DAPI for 10 min and again washed 3 times with PBS. Images were taken on the Cytation 1 cell imaging multimode reader (BioTek) as described above.

### DNA deamination activity assays.

First, 250,000 293T cells were seeded into a 6-well plate in the complete media and allowed to adhere overnight. The next day, each well was transfected with 1 μg of each A3B expression construct or EGFP control. At 48 h posttransfection, the supernatant of each well was removed, and cells were resuspended in 1 mL growth media. The resuspended cells from each well were removed from the plate and split evenly between two 1.5 mL Eppendorf tubes (500 μL each), one for immunoblot and one for deaminase activity. All samples were centrifuged at 8,000 rpm for 5 min and supernatant discarded. The set of samples for immunoblot were resuspended in 50 μL 2× reducing sample buffer (RSB) for later western blot of protein expression. The samples used for deaminase activity were resuspended in 100 μL of modified HED buffer (25 mM HEPES, 15 mM EDTA, 10% glycerol, 1 tablet of Sigma-Aldrich complete protease inhibitor cocktail) and immediately placed on ice. Then, these samples were sonicated for three 5-s pulses at the lowest setting, placed on ice between each pulse, and centrifuged at 20,000 *g* for 20 min at 4°C. The soluble lysate was transferred to a new, prechilled 1.5-mL Eppendorf tube. Next, 10 μL of soluble lysate was incubated at 37°C for 2 h with 10 μL reaction mix containing a fluorescently labeled oligonucleotide (0.25 μL RNase A, 800 nM oligonucleotide, 10× UDG buffer, 0.25 μL UDG, 1.0 μM oligonucleotide) for a total reaction volume of 20 μL. The fluorescently labeled oligonucleotide used contains only one potential A3B consensus target cleavage site (5′-ATTATTATTATTCAAATGGATTTATTTATTTATTTATTTATTT-fluorescein-3′). A no-lysate control was used to demonstrate the baseline level of uncleaved oligonucleotide in each sample (Mock). EGFP-transfected cell lysate was used as a control to demonstrate baseline levels of deaminase activity due to endogenous expression of deaminase enzymes. After the 2-h incubation, 100 mM NaOH was added to each reaction and incubated at 98°C for 10 min. Then, 2× formamide buffer (80% formamide, 1× Tris-borate-EDTA (TBE), 0.05% bromophenol blue, 0.01% xylene cyanol) was added to each reaction, mixed by pipet, and incubated at 98°C for 5 min. The samples were run through a 15% Urea-TBE gel at 12 W for 45 min. A Typhoon FLA-7000 biomolecular imager (GE Healthcare) on fluorescence mode was used to image the gel and detect deaminase activity.

### Immunoblots.

All protein samples were run on a Criterion 4 to 20% SDS-PAGE Tris-HCl gel (Bio-Rad) and transferred to a 0.2 μm nitrocellulose membrane (Bio-Rad Inc. no. 1620112) using the Power Blotter XL system (Invitrogen). EGFP fusion proteins and loading controls were detected on a nitrocellulose membrane with a rabbit anti-EGFP antibody (Abcam, ab290; 1:5000), mouse anti-GAPDH (Abcam, ab9484; 1:1000), or mouse anti-HSP90 (BD Biosciences, bd610418; 1:1000). Blots were developed using anti-mouse 680LT and anti-rabbit 800CW secondary antibodies (LiCor) and imaged on an Odyssey Fc infrared fluorescent scanner (LiCor).

### NES and NLS inhibitor experiments.

First, 50,000 HeLa were seeded into a 24-well plate (Sigma-Aldrich no. Z707805) in complete media and allowed to adhere overnight. The next day, each well was transfected with 200 ng of human A3B-EGFP, rhesus A3B-EGFP, or AID-EGFP. Two days posttransfection, cells were treated for 180 min with 12.5 nM Leptomycin B (CRM1/NES inhibitor), 25 μM ivermectin (IMPORTIN-alpha/NLS inhibitor), or vehicle control. Cells were fixed with 4% formaldehyde in phosphate-buffer saline (FA-PBS) for 15 min at room temperature. DAPI was used to stain the nucleus. Cells were imaged at ×20 magnification on a Cytation 1 cell imaging multimode reader (BioTek) with the following LED cubes: DAPI (377/447) and EGFP (469/525).

### Statistical analyses.

GraphPad Prism 9.0 was used for statistical analysis of quantitative data. Outliers were removed using ROUT analysis (Q = 1%). Quantitative data were represented as mean +/− SEM of the nuclear-to-cytoplasmic (N:C) ratio.
