# Supplementary figures and images for: Ancestral APOBEC3B Nuclear Localization Is Maintained in Humans and Apes and Altered in Most Other Old World Primate Species
Source: mSphere. 2022 Nov 14;7(6):e00451-22. doi: 10.1128/msphere.00451-22 (PMC9769932; doi:10.1128/msphere.00451-22)

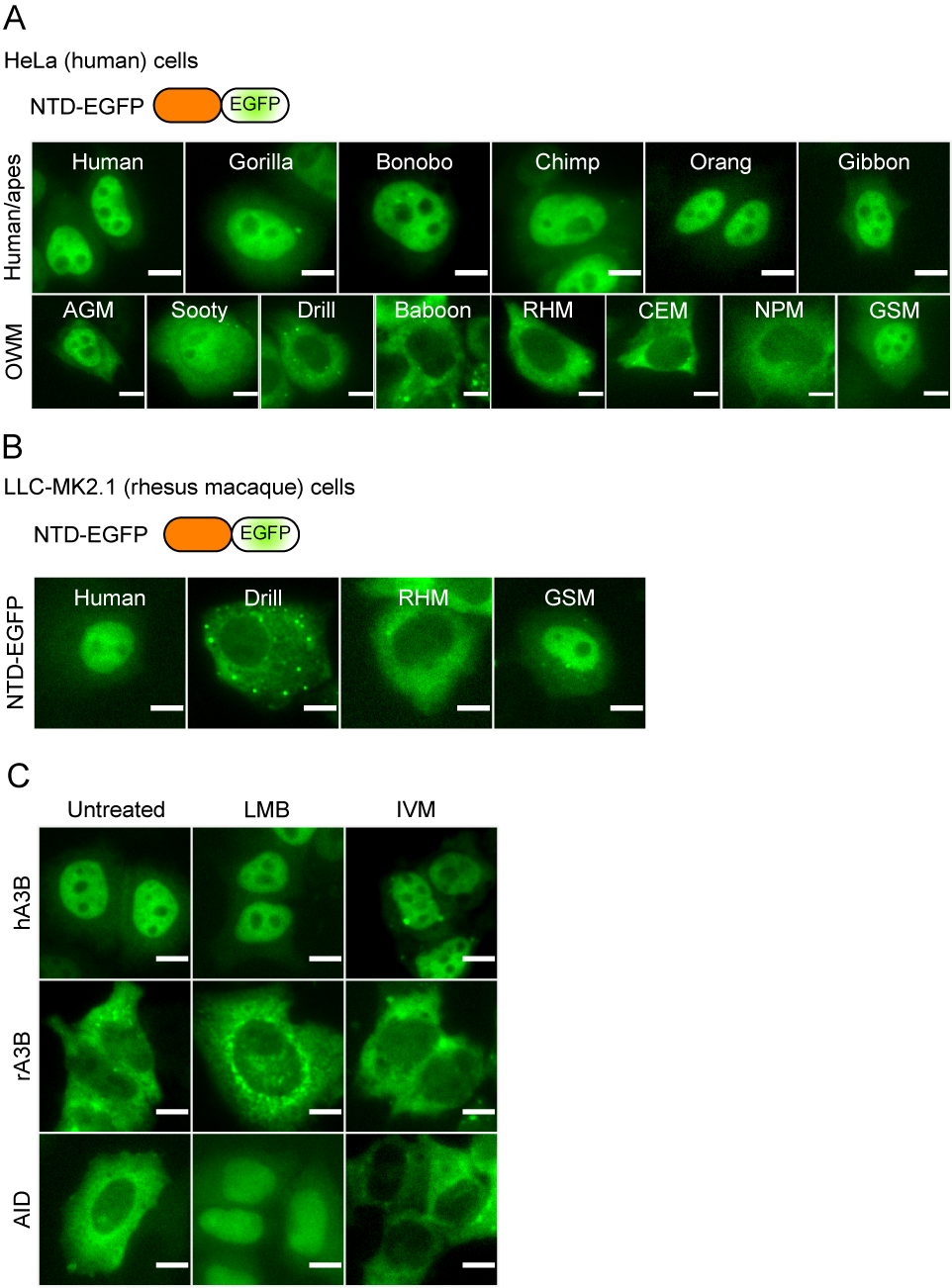

Supplement: FIG S3 [file msphere.00451-22-s0003.tif]

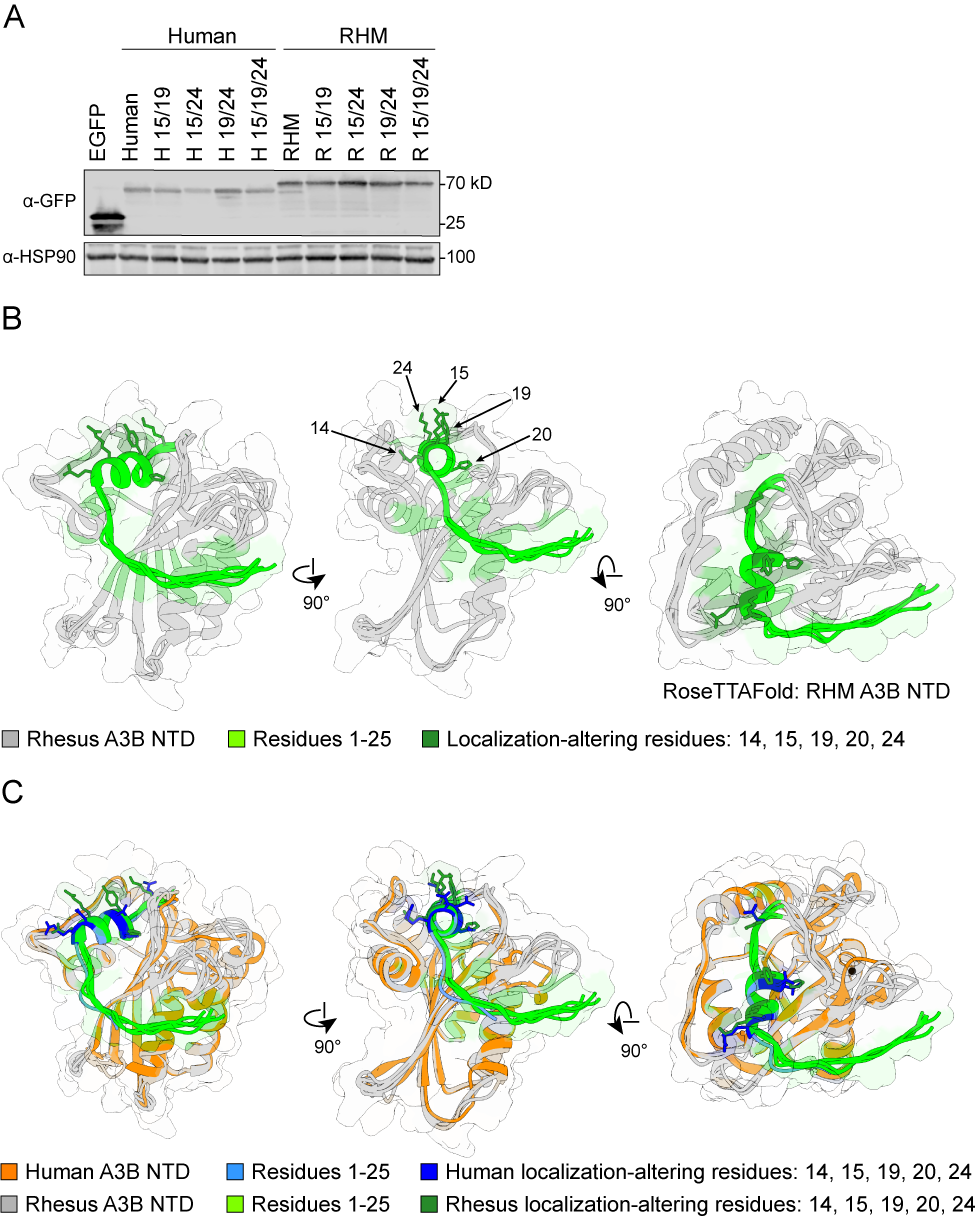

Supplement: FIG S4 [file msphere.00451-22-s0004.tif]
